# Personalized Fair Split Learning for Resource-Constrained Internet of Things

**DOI:** 10.3390/s24010088

**Published:** 2023-12-23

**Authors:** Haitian Chen, Xuebin Chen, Lulu Peng, Yuntian Bai

**Affiliations:** 1 College of Science, North China University of Science and Technology, Tangshan 063210, China; chenht@stu.ncst.edu.cn (H.C.);; 2 Hebei Key Laboratory of Data Science and Application, Tangshan 063210, China; 3 Tangshan Key Laboratory of Data Science, Tangshan 063210, China

**Keywords:** Internet of Things, federated learning, split learning, personalized model, data heterogeneity, collaborative fairness

## Abstract

With the flourishing development of the Internet of Things (IoT), federated learning has garnered significant attention as a distributed learning method aimed at preserving the privacy of participant data. However, certain IoT devices, such as sensors, face challenges in effectively employing conventional federated learning approaches due to limited computational and storage resources, which hinder their ability to train complex local models. Additionally, in IoT environments, devices often face problems of data heterogeneity and uneven benefit distribution between them. To address these challenges, a personalized and fair split learning framework is proposed for resource-constrained clients. This framework first adopts a U-shaped structure, dividing the model to enable resource-constrained clients to offload subsets of the foundational model to a central server while retaining personalized model subsets locally to meet the specific personalized requirements of different clients. Furthermore, to ensure fair benefit distribution, a model-aggregation method with optimized aggregation weights is used. This method reasonably allocates model-aggregation weights based on the contributions of clients, thereby achieving collaborative fairness. Experimental results demonstrate that, in three distinct data heterogeneity scenarios, employing personalized training through this framework exhibits higher accuracy compared to existing baseline methods. Simultaneously, the framework ensures collaborative fairness, fostering a more balanced and sustainable cooperation among IoT devices.

## 1. Introduction

In recent years, with the rapid advancements in the Internet of Things (IoT) and distributed computing driven by big data, IoT devices have been extensively deployed. Particularly in the Industrial Internet of Things (IIoT) domain, a multitude of sensor devices are dispersed across various locations for the purpose of network data collection. However, aggregating network big data to centralized servers may potentially infringe upon the data privacy of individual enterprises or clients, potentially leading to the formation of multiple data silos [1]. To address the challenges stemming from the development of IoT and the application-related “data silos” phenomenon, federated learning (FL) [2] has gained increasing attention as a distributed learning method. FL allows multiple participants to collaboratively train a shared global model without sharing raw data, thus ensuring the protection of participant data privacy. However, in IoT environments, some resource-constrained IoT devices cannot easily train complex local models using traditional FL methods due to limitations in computational and storage resources.

Split learning (SL), as an evolved form of FL, proves to be more suitable for resource-constrained IoT devices [3]. SL allows one to split complex client-side local models, offloading most of the computational tasks of the model to a central server while retaining only subsets of the model at the client end. This method effectively reduces the computational and storage burden on IoT devices, making them more adaptable to resource-limited environments. In addition, in complex IoT environments, there are also issues of data heterogeneity and uneven distribution of benefits among devices. On the one hand, data heterogeneity manifests as each device being influenced by its environment and individual preferences, resulting in a unique data distribution and significant differences in data volume. For instance, in the realm of sensors, distinct sensor devices exhibit unique data distributions and noticeable disparities in data volume due to differences in sensing capabilities and deployment environments. Due to data heterogeneity, models trained through SL may have difficulty in effectively capturing common features of different data types across devices, which may lead to the degradation of the model’s performance on a specific device or even prevent affected devices from participating in SL training [4]. On the other hand, the uneven distribution of benefits emerges due to imbalances in data quality and scale among devices. Some smaller-scale IoT entities, despite having lesser data volume, possess more representative or higher-quality data, playing a crucial role in model training. However, despite their substantial contributions, these entities often receive less benefit compared to larger-scale entities [5]. Such an imbalance might hinder these smaller entities from achieving expected outcomes during the training process, prompting them to opt out of cooperation, leading to the termination of SL training. Hence, in complex IoT environments, a mechanism is needed to address the issues encountered in SL training, catering to device-specific requirements while ensuring fairness.

To address the aforementioned issues, a personalized and fair split learning framework, termed Split Learning with Personalized Fairness (SplitLPF), has been proposed. The framework begins by splitting the model into a three-segment U-shaped structure during model training. In this structure, the central server acts as the offloading entity, undertaking the burden of intensive computational tasks to facilitate the training of intricate models on resource-constrained client devices. Simultaneously, client devices retain personalized model subsets locally to cater to the specific individualized needs of different clients, enhancing their ability to address the challenges posed by data heterogeneity. Furthermore, during the model-aggregation phase, the central server adopts an optimized aggregation weight methodology. This method ensures fair benefit distribution among various clients in federated learning by reasonably allocating model-aggregation weights based on client contributions, utilizing estimations derived from differences in dataset sizes and gradient directions. This approach incentivizes the active participation of contributors in the model-training process, thereby achieving collaborative fairness.

The main contributions of this paper are as follows:

(1) This paper proposes a personalized U-shaped split architecture, designed to fulfil specific individualized requirements of different clients in complex IoT environments while effectively training sophisticated models on resource-constrained client devices;
(2) This paper introduces a model-aggregation method based on contribution estimation. By estimating the contribution size of each client, the aggregation weights of the model are reasonably assigned to ensure a fairer distribution of benefits among different clients;
(3) The experimental results demonstrate that our proposed framework not only exhibits high accuracy in its personalized training approach under different data heterogeneity scenarios, it also ensures fairness in collaboration and promotes more balanced and sustainable cooperation among clients.

## 2. Background and Related Work

### 2.1. Distributed Learning in the IoT

In the realm of the IoT, distributed learning has emerged as a pivotal technology, offering effective solutions for handling large-scale dispersed sensor and device data. In this context, distributed learning methods such as federated learning and split learning and their variants play significant roles in IoT [6]. These methods underscore privacy preservation [7], decentralization [8], and resource efficiency [9], holding promise to propel the development of IoT, fostering intelligent decision-making and enabling the realization of data-driven applications.

#### 2.1.1. Federated Learning

**Figure 1:**
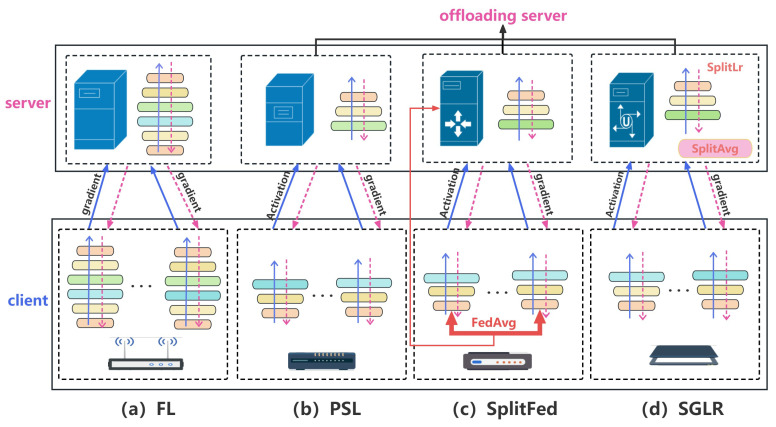
Comparison of distributed learning. (**a**) Federated Learning; (**b**) Parallel Split Learning; (**c**) SplitFed learning; (**d**) SGLR.

In an FL system, two primary entities can be divided: there are the data owners collaborating in model training, referred to as clients, and there is the model owner who is responsible for coordinating the training process and model aggregation, termed the central server [10]. Let N={1,2,...,K} represent a set containing *K* clients, each of which possesses its local dataset $D_{k}\left(k\in K\right)$, constituting the entire dataset $D=\cup_{k=1}^{K}D_{k}$. The FL system is shown in Figure 1a. The FL system in the initial phase of model training, and the central server first initializes the model and broadcasts the initial global model to the selected clients. Subsequently, each selected client employs its local dataset to train its local model, uploading the trained model parameters (gradients) to the central server. The central server aggregates all received client gradients, producing a new global gradient, ultimately using this global gradient to update all clients’ models. This local training and global aggregation process iterate in a loop until the global model converges, thereby completing the entire model-training process. However, some resource-constrained IoT devices face challenges in effectively utilizing traditional federated learning methods to train complex local models.

#### 2.1.2. Split Learning and Its Variants

To address the challenge of resource constraints, SL proposes to use servers to collaboratively train local models without the need to scale down the model and obtain raw data from the client [11]. SL involves dividing the complete model ω into client-side $\omega_{C}$ and server-side models $\omega_{S}$. The parallel split learning (PSL) system is shown in Figure 1b, where each client performs forward propagation steps in parallel during PSL training until a specific clipping layer is reached. The output of this cut layer is then transmitted to the server, where the remaining part of the model undergoes forward propagation to generate prediction results. The training loss is computed on the server using labels and predicted values. Subsequently, a backward propagation step is performed on the server until it reaches the cut layer [12]. Afterwards, the gradients are sent back to the respective clients, enabling parallel execution of backward propagation on the first segment of the model (the client-side model). This process iterates until the final model is obtained. In this configuration, there is no need for data or weight aggregation to accomplish the training of complex models. However, in certain configurations, appropriate variants of SL are applied, some of which involve operations such as aggregating model weights and aggregating intermediate data [13,14,15]. In weight aggregation-based SL, clients transmit their local model weights to a trusted third party for the aggregation of these weights. For instance, in SplitFed learning [13] as illustrated in Figure 1c, the model is divided into two segments and trained in a manner similar to PSL. After each client completes a training round, its local model weights are transferred to the server, which employs the FedAvg aggregation method to integrate these local models. Joshi et al. [14] proposed the SplitFed learning framework SFPL with positive labels to improve the deep learning model training for resource-constrained IoT clients. In intermediate data aggregation-based SL, intermediate data like activations or gradient information from different clients are aggregated. For instance, in SGLR [15] shown in Figure 1d, the server aggregates local gradients from clients instead of model parameters. Additionally, SGLR divides the learning rate into server-side and client-side learning rates, adjusting them separately to support parallelism among multiple clients. SGLR effectively reduces the volume of shared information among clients and mitigates client decoupling issues [16].

The model partitioning employed in the aforementioned SL and its variants has somewhat increased communication costs due to the transmission of intermediate data during the model-training process, which needs to be exchanged in each iteration, resulting in higher communication overhead. To mitigate the communication cost in SL, Chen et al. [17] proposed an asynchronous training scheme based on loss, where gradients are transmitted at specified intervals and quantized using 8-bit floating-point before transmission, thereby reducing the communication cost of SL. Oh et al. [18] introduced a communication-efficient SL framework called SplitFC, utilizing adaptively compressed intermediate features and gradient vectors based on varying dispersion levels in vectors. They combined adaptive feature dropping and adaptive feature quantization as compression strategies to minimize communication overhead. Lin et al. [19] presented an efficient PSL framework, EPSL, which aggregates the last layer’s activation gradients during backpropagation. They conducted joint optimization on EPSL’s subchannel allocation, power control, and layer pruning to reduce communication costs. While most methods for reducing communication costs adopt techniques like value quantization and feature compression, these may significantly impact the accuracy of the global model. However, in [20], the number of communications is reduced and the model quality is improved by transmitting more information and smaller synthetic data. This paper is inspired by that, and lightweight synthetic data transfer is chosen to alleviate some communication pressure.

### 2.2. Distributed Learning Challenges and Strategies in IoT

#### 2.2.1. Challenges to Distributed Learning in the IoT

In the complex IoT environment, SL model splitting proves effective at addressing resource-constrained challenges. However, the complexity and specificity of the IoT environment poses additional challenges, notably data heterogeneity and uneven benefit distribution. These challenges hold significant importance in both academic research and practical applications.

*Data Heterogeneity*

In IoT environments, particularly in IIoT environments, the diversity among different sensor devices, arising from variations in sensing capabilities, environmental conditions, and data characteristics, results in significant differences in collected data. This diversity poses challenges for traditional distributed learning methods, which commonly operate under the assumption of data being identically and independently distributed (IID). Consequently, there is a pressing need to explore novel methodologies to address the issue of data heterogeneity based on the assumption of non-independent and identically distributed (Non-IID) data.

*Uneven Benefit Distribution*

The imbalance in data quality and quantity among different devices results in varied benefits obtained from their contributions. However, fairness becomes particularly crucial when engaging in data sharing and collaboration among multiple sensor devices. Given that these devices are typically distributed across different locations and collect diverse types of data, an effective collaborative approach is required to achieve integrated sensing and analysis. Therefore, there is a need for fair model aggregation so that the contribution of each device is justly recognized, thus effectively improving the problem of unequal distribution of benefits and promoting a more balanced cooperative relationship.

#### 2.2.2. Strategies to Distributed Learning in the IoT

In this subsection, we aim to review the representative techniques (strategies) proposed to address the typical challenges of distributed learning in the IoT context. Given the focal point of this paper on personalized fair SL, and the fact that SL-related work has been discussed in detail earlier, this subsection will provide the necessary background information on personalized processing and fairness design.

*Personalized Processing*

To address the challenges posed by the non-IID data, an effective strategy involves personalized handling at both the data and model levels to mitigate data heterogeneity and deliver high-quality personalized models for each client. Personalized FL encompasses various approaches, including those based on data, models, and architecture [21]. Data-based methods aim to reduce the heterogeneity in client data distributions to alleviate client drift issues. This approach can be realized using techniques such as data enhancement [22] and client selection mechanisms [23]. Model-based approaches aim to learn a robust global FL model while catering to personalized services for each individual client. This can be achieved by learning global and local features of each client [24], leveraging historical memory to learn global representations [25], and improving local models through transfer learning [26]. Architecture-based methods focus on providing tailored and personalized model architectures for individual clients. The approach can be implemented using techniques such as parameter decoupling [27] and knowledge distillation [28], where parameter decoupling mainly provides a personalization layer for each client.

In the realm of personalized SL, relatively fewer researchers have delved into this area. Among them, Wadhwa et al. [29] proposed that PFSL, which employs transfer learning for pre-training. Subsequently, by freezing certain shared layer weights and updating the unfrozen ones, each client can train their model for varying numbers of epochs, thereby enabling personalized operations. Han et al. [30] proposed SplitGP, leveraging multi-output neural networks to capture both generalized and personalized learning requirements. This approach empowers client models to optimize for their respective primary tasks during the training process, fostering robust personalized capabilities. However, the aforementioned personalized SL methods predominantly focus on model-based approaches. In this paper, we aim to adopt an architecture-based approach. We intend to meet customized personalized requirements through the design of foundational and personalized model architectures.

*Fairness Design*

Fairness design can effectively address the challenges posed by uneven benefits distribution. In this context, both model fairness and collaborative fairness have garnered significant attention as distinct perspectives in fairness design. Model fairness concerns whether the global model constructed in federated learning maintains fairness in its treatment of different participants [31], aiming to enhance uniformity in performance across all involved parties. On the other hand, collaborative fairness places greater emphasis on the cooperative interaction among participants in federated learning. This entails ensuring equal opportunities for engagement and contribution among all participants during training, alongside fair treatment of each participant’s contributions [32].

For model fairness, Hu et al [33] proposed the FedMGDA+ method, which performs multi-objective optimization by optimizing the loss function of each FL client individually and simultaneously to avoid sacrificing the model performance of any client. Cui et al. [34] proposed a constrained multi-objective optimization framework, learning a model that satisfies the fairness constraints of all clients with consistent performance by optimizing the agent’s maximal function involving all objectives. Li et al. [35] proposed the Ditto method, employing inter-client fine-tuning to minimize individual losses significantly. They augmented this by adding regularization terms, bringing personalized models closer to the optimal global model to achieve consistent model performance. In contrast to model fairness, collaborative fairness garners higher attention due to its direct involvement with collaboration and contributions among clients. Lyu et al. [36] proposed a collaborative fairness framework using a reputation mechanism to gauge participant contributions, converging toward different models to ensure fairness. Xu et al. [37] designed a gradient-reward mechanism, in which fairness is ensured by sparsification to treat the aggregated gradient downloaded by the server as an expected marginal contribution to each client. However, in most collaborative fairness designs, more or less shapely values [38] were used to participate in the contribution assessment, resulting in high computational cost. Therefore, in this paper an approximation is used to measure the contribution size to alleviate the computational pressure.

## 3. SplitLPF Frame

**Figure 2:**
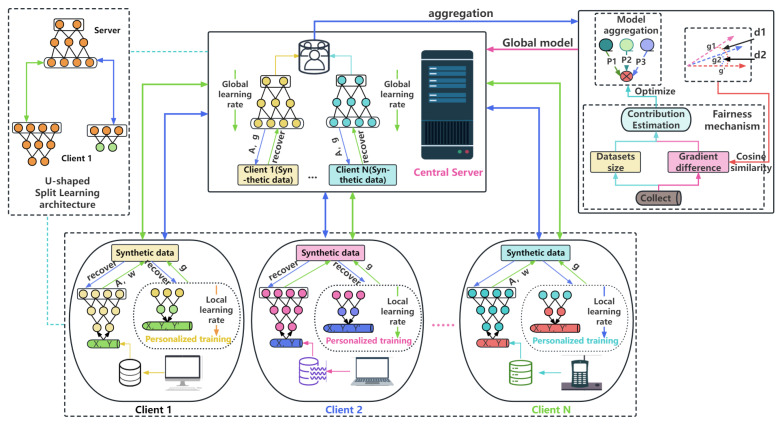
SplitLPF framework.

The paper introduces a personalized and fair SL framework termed SplitLPF, as illustrated in Figure 2. In this framework, clients split the model and offload intricate computational tasks to a central server; this flexible splitting enables tasks to be assigned based on the characteristics of the device. During the model-training process, clients and the central server collaboratively execute the foundational model training. Subsequently, clients engage in more flexible personalized model training. This personalized training helps to improve the accuracy and performance of devices (such as sensors), making them better adapted to specific application scenarios and environments. During the model-aggregation process, model weights are optimized based on client contribution estimations, enabling the aggregation of split models. This ensures fairness among participants, providing an incentive for active participation in model training. To minimize communication overhead, the framework adopts a lightweight synthetic data transmission method. The SplitLPF framework amalgamates the strengths of both the central server and clients, effectively leveraging resources to address challenges faced by resource-constrained devices in complex IoT environments. Simultaneously, it ensures fairness in model training.

The design of the SplitLPF framework is divided into three components: model partition, personalized training, and fairness mechanism guarantee. Subsequently, detailed introduction will be provided for each of these components.

### 3.1. Model Split

In SL model training, it is common to split the model into two segments: the client-side model $\omega_{C}$ and the server-side model $\omega_{S}$. The bulk of computational tasks is offloaded to the server-side model $\omega_{S}$ to better accommodate the model training requirements of resource-constrained devices. This approach simultaneously reduces the need for transmitting sensitive data, consequently lowering the risk of client data exposure.

Consider a system with a single central server and *N* clients; each client has its own private dataset (local dataset) $\left\{D_{1},D_{2},...,D_{N}\right\}$, and $D_{k}$ denotes the dataset owned by the *k*-th participant. Similar to FL model training, the goal of SL training is to collaboratively determine an optimal model $\omega^{*}$ through the cooperation of clients and the central server, fulfilling the minimization of the loss function as shown in Equation (1):

(1) $$\min_{\omega }\;F\left(\omega \right)=\min_{\omega }\;\sum_{k=1}^{N}p_{k}F_{k}\left(\omega \right)$$

where $p_{k}$ denotes the weight of participant *k* local model when aggregated at the central server, satisfying $p_{k}\geq 0$ and $\sum_{k=1}^{N}p_{k}=1$. It is commonly set as $p_{k}=\frac{|D_{k}|}{\sum_{k=1}^{N}|D_{k}|}$, where $F_{k}\left(\omega \right)$ denotes the local loss function of participant *k*. This function is further defined as the sum of the loss functions between the client-side model $\omega_{C}$ and the server-side model $\omega_{S}$, as illustrated by Equation (2):

(2) $$F_{k}\left(\omega \right)=\frac{1}{|D_{k}|}\sum_{x\in D_{k}}(\ell_{C}\left(x;\omega_{C}\right)+\ell_{S}\left(A_{k};\omega_{S}\right))$$

where $\ell_{C}\left(\cdot \right)$ and $\ell_{S}\left(\cdot \right)$ denote the loss functions of the client-side models and server-side models, respectively. $A_{k}$ denotes the intermediate result (such as activation), $D_{k}$ denotes the client-side dataset, and *x* denotes the local data. In SL, clients train the model up to its splitting layer using raw data and then transmit the intermediate results (activation data $A_{k}$) from this splitting layer to the server. The server employs the received intermediate results from clients to train the remaining layers of the model, completing the forward propagation of the model. Subsequently, the server conducts backpropagation up to the splitting layer and forwards the intermediate results (gradient *g*) to the clients. Utilizing the gradient, each client performs its backpropagation on the split model, iteratively updating it to accomplish the entire model training. Each client *k* updates the client-side model based on the local gradient information as shown in Equation (3):

(3) $$\omega_{C,k}^{t+1}=\omega_{C,k}^{t}-\eta^{t}\nabla F_{C,k}\left(\omega_{C,k}^{t}\right)$$

where $\omega_{C,k}^{t}$ denotes the local model of client *k* in round *t*, $\eta^{t}$ denotes the learning rate in round *t*, and $\nabla F_{C,k}\left(\cdot \right)$ denotes the gradient of the local model of client *k*. The server updates the server-side model according to Equation (4):

(4) $$\omega_{S,k}^{t+1}=\omega_{S,k}^{t}-\eta^{t}\nabla F_{S,k}\left(\omega_{S,k}^{t}\right)$$

where $\omega_{S,k}^{t}$ denotes the server model updated for round *t* based on the intermediate results of client *k*, and $\nabla F_{S,k}\left(\cdot \right)$ denotes the gradient of the server model. In resource-constrained environments, some devices may have difficulty in transmitting large volumes of data (such as activations) for long periods of time due to unstable network connections, limited bandwidth, and low device performance, and thus they may not be able to effectively participate in model training. However, in this paper we address the communication pressure by substituting bulk data transmission with lightweight synthetic data. In distributed learning, model compression primarily occurs at two main places: the local model update transmission from the client to the server in the upstream, and the global model update transmission from the server to the client in the downstream [39]. Similar to compression work, we replace model updates with synthetic data in both upstream and downstream data transmission. In the conventional SL model training, $A_{k}$ is upstream transmission data, $\nabla F_{S,k}\left(\cdot \right)$ is downstream transmission data, and clients k and servers attempt to find synthetic data $D_{Ck}^{syn}$ and $D_{Sk}^{syn}$ as replacements for transmission data. The most intuitive approach is to minimize the distance metric between the model generated using synthetic data and the model generated from real data [40]. Therefore, we optimize the synthetic data using the objective function of the $\ell_{2}$ distance between simulated model weights and real model weights, employing stochastic gradient descent (SGD) to generate appropriate lightweight synthetic data $D^{syn}$, as shown in Equation (5):

(5) $$D^{syn}\leftarrow SGD\left(D^{syn};\nabla f\left(\omega^{raw};\omega^{syn}\right)\right)$$

where $\omega^{syn}=M\left(D^{syn};\omega \right)$ denotes the model generated using synthetic data, M(⋅) denotes the model update function, and $\omega^{raw}$ denotes the model generated using real data. ∇f(⋅) denotes the loss function based on the $\ell_{2}$ distance. Subsequently, the server involved in upstream communication and the client involved in downstream communication can use this synthetic data to recover local activation data or gradient information. The server and client use the same process as the generation of $D^{syn}$ to recover the original data $D^{raw}$. This communication scheme has been widely applied in the literature [40,41,42,43], and specific details are not further elaborated on in this paper. In comparison to large batches of real data, lightweight synthetic data, while having the same input dimensions, achieves similar feature representations to large batches of data in smaller batches and can be recovered as real data with less computation. Based on the experimental observations in Section 4.4, we find that the use of lightweight synthetic data transmission reduces the communication cost by almost approximately 24% compared to the existing personalized fair SL.

### 3.2. Personalized Training

In complex IoT environments, model partitioning effectively alleviates the computational burden on resource-constrained clients and enhances prediction accuracy to some extent. However, in IoT environments, particularly in IIoT domains, various types of sensor devices may possess different sensing capabilities, disparate data distributions, and varying data volumes, exacerbating the problem of data heterogeneity. This data heterogeneity issue can potentially impact model performance. In extreme scenarios, data types, data distributions, or even data contents among different sensor devices might be entirely dissimilar. Under such circumstances, it is difficult to use SL model training methods to handle this heterogeneity effectively. In this context, personalized training becomes paramount.

In the model training phase, this paper further subdivided the traditional SL to form a three-stage U-shaped structure. The client-side retains the input and output layers locally and performs the computation of the loss function, while the central server performs the complex hidden layer computation. The specific process of training is as follows: during each global iteration, individual clients compute the first part (front part) of the model and upload the intermediate data to the central server in parallel. Leveraging more robust computational capabilities, the central server swiftly conducts corresponding forward propagation, completing the second part (middle part) of the model training. During this period, the clients and server collaborate to achieve the generalization phase of model training. Subsequently, the central server sends the results back to the client, which executes the SGD optimization algorithm to compute the prediction results, loss values, and gradients based on the respective local learning rates to complete the personalization phase training (back part) locally. Then, clients utilize the gradients to update their local parameters and transmit them back to the central server. The central server uses the gradient results to update the intermediate model parameters and sends the results back to the clients. Finally, the clients update the parameters of the first part of the model. In this process, clients and servers first complete the base model training, after which the clients independently execute personalized model training aimed at optimizing the model to better adapt to the characteristics of diverse devices, thereby enhancing the model’s performance and adaptability. Throughout the personalized training, clients retain label data locally, preventing other clients and servers from inferring model specifics or local data through label data. This ensures the enhancement of model training efficiency while safeguarding data privacy.

The client-side model $\omega_{C}$ training can be further broken down into two components: base model training $\omega_{CB}$ and personalized model training $\omega_{CP}$. Each round of update of the base model and personalized model is represented as Equations (6) and (7):

(6) $$\omega_{CB,k}^{t+1}=\omega_{CB,k}^{t}-\eta^{t}\nabla F_{CB,k}\left(\omega_{CB,k}^{t}\right)$$

(7) $$\omega_{CP,k}^{t+1}=\omega_{CP,k}^{t}-\eta_{k}^{t}\nabla F_{CP,k}\left(\omega_{CP,k}^{t}\right)$$

where $\nabla F_{CB,k}\left(\cdot \right)$ and $\nabla F_{CP,k}\left(\cdot \right)$ denote the model gradient for client *k* base model and personalized model training, respectively. $\eta_{k}^{t}$ is the local learning rate of client *k* in round *t*, and $\eta^{t}$ denotes the global learning rate in round *t*. During personalized training, the pseudo-code for the client to perform the model update locally is shown in Algorithm 1.

### 3.3. Fairness Mechanism

Personalized training exhibits significant advantages in addressing data heterogeneity issues in complex IoT environments. However, in the typical model-training process, the aggregation of model parameters typically relies on the dataset’s size to allocate weights for achieving a weighted average model aggregation. This approach may lead to the uneven distribution of benefits, making it challenging for smaller-scale devices possessing high-quality data to achieve expected outcomes during model training. This leads to smaller devices opting out of the collaboration and terminating model training. This poses a serious threat to the process of collaborative learn as the contribution of each device is not negligible. To address this challenge, the introduction of mechanisms for collaborative fairness is necessary.

Collaborative fairness aims to ensure the fair recognition of each device’s contribution. However, the size of the dataset by itself is insufficient to provide enough information to accurately reflect the contribution of each local client. Therefore, relying solely on dataset size-based weighted aggregation might not be the optimal aggregation strategy.
**Algorithm 1** ClientUpdate**Input:** $D_{k}$: local dataset; *N*: number of clients; $\eta^{t}$: global learning rate; $\eta_{k}^{t}$: local learning rate; $Y_{k}$: the true labels;**Output:** $\omega_{CP,k}^{t}$: personalized model; $\omega_{CB,k}^{t}$: base model;1:**Procedure** ClientForwordProp($\omega_{CB,k}^{t}$):2:**if** epoch t = 0 **then**3:    Set the local activations $A_{CB,k}^{t}=⌀$;4:**else**5:    $D_{Sk}^{syn}\leftarrow$ServerUpdate()// Receive data transmitted by the server;6:    Recover the global ${\hat{\omega }}_{CB}^{t}$ based on $D_{Sk}^{syn}$;7:**end if**8:Forward propagate the local data $D_{k}$ to the $\omega_{CB,k}^{t}$ cutting layer and obtain the local activations $A_{CB,k}^{t}$;9:Generate synthetic data $D_{Ck}^{syn}$ based on $A_{CB,k}^{t}$, $|D_{k}|$, and $\omega_{CB,k}^{t}$;10:Send the synthetic data $D_{Ck}^{syn}$ to the central server;11:Wait for ClientBackProp($\omega_{CP,k}^{t}$) to complete;12:**end procedure**13:**Procedure** ClientBackProp($\omega_{CP,k}^{t}$):14:$D_{Sk}^{syn}\leftarrow$ServerUpdate() // Receive data transmitted by the server;15:Recover $A_{S,k}^{t}$ and $dA_{S,k}^{t}$ based on $D_{Sk}^{syn}$;16:**if** state = Personalized training **then**17:    Forward propagation with $A_{S,k}^{t}$ on $\omega_{CP,k}^{t}$;18:    Calculate ${\hat{Y}}_{k}$ and loss calculation with $Y_{k}$ and ${\hat{Y}}_{k}$;19:    Back-propagation calculate $\nabla \ell_{C}\left(\omega_{CP,k}^{t}\right)$;20:    Generate $D_{Ck}^{syn}$ using $dA_{CP,k}^{t}:=\nabla \ell_{C}\left(A_{S,k}^{t};\omega_{CP,k}^{t}\right)$ and send it to the server;21:    Update the personalized model $\omega_{CP,k}^{t+1}\leftarrow \omega_{CP,k}^{t}-\eta_{k}^{t}\nabla \ell_{C}\left(\omega_{CP,k}^{t}\right)$;22:**else**23:    Calculate $\nabla \ell_{C}\left(\omega_{CB,k}^{t}\right)$ using $dA_{S,k}^{t}$;24:    Update the base model $\omega_{CB,k}^{t+1}\leftarrow \omega_{CB,k}^{t}-\eta^{t}\nabla \ell_{C}\left(\omega_{CB,k}^{t}\right)$;25:**end if**26:**end procedure**

In this case, a viable aggregation strategy involves fairly distributing benefits by considering the contribution levels of the clients comprehensively. It might be beneficial to incorporate the discrepancy between local clients and the global model into the consideration for benefit distribution. Intuitively, certain clients with high-quality data provide more trustworthy and informative gradient information, enabling the model to update parameters more accurately and converge faster towards the global optimum, which results in a model whose gradient direction is closer to that of the global model. Consequently, clients whose gradient direction is more similar to that of the global model might contribute more significantly to the global model. This implies that the contribution level of clients might be related to the similarity of their gradient direction with the global model’s gradient direction. Hence, gradient direction disparity could serve as a useful supplementary metric. In other words, considering both dataset size and gradient direction disparity for assessing contribution levels seems more comprehensive. Section 3.5 provides theoretical analysis about this; it promotes fairness in benefit distribution through contribution estimation. The measurement of fairness could be defined as follows:

**Definition 1.**  *If the test performance distribution of model $\omega_{1}$ is more uniform than that of model $\omega_{2}$, which is expressed as: $std\left(F_{k}\left(\omega_{1}\right)\right)<std\left(F_{k}\left(\omega_{2}\right)\right)$, it is said that model $\omega_{1}$ is fairer than model $\omega_{2}$, where $F_{k}\left(\cdot \right)$ represents the test loss on client k, and std(⋅) denotes the standard deviation.*

The specific implementation steps of the fairness mechanism based on contribution estimation are as follows.

First, in order to reflect the gradient direction discrepancy, cosine similarity is used for measurement. In the *t*-th round, the discrepancy between client *k* and the global model is as shown in Equation (8):

(8) $$d_{k}=cos\left(\nabla F_{k}\left(\omega_{k}^{t}\right),\nabla F\left(\omega^{t-1}\right)\right)$$

where $\nabla F_{k}\left(\omega_{k}\right)$ denotes the local gradient of client *k*, and ∇F(ω) denotes the global gradient. The global gradient can be expressed as: $\nabla F\left(\omega \right)=\sum_{k=1}^{N}p_{k}\nabla F_{k}\left(\omega_{k}\right)$, where $p_{k}$ denotes the aggregation weights of the model.

Then, the discrepancy values are further normalized as shown in Equation (9):

(9) $${\bar{d}}_{k}=\frac{|d_{k}|}{\sum_{k=1}^{N}|d_{k}|}$$

Finally, the aggregation weights of the model are optimized according to the size of the client’s contribution, and the optimized aggregation weights are as shown in Equation (10):

(10) $$p_{k}=\alpha n_{k}+\left(1-\alpha \right){\bar{d}}_{k}$$

where α is a hyperparameter and $n_{k}=\frac{|D_{k}|}{\sum {k=1}^{N}|D_{k}|}$ denotes the dataset size. In Equation (10), there is a free-rider attack by some clients that are small and in the opposite direction of the global model training, making the aggregation weights negative, and this can be circumvented using the ReLU function. Therefore, it can be normalized as shown in Equation (11):

(11) $$p_{k}=\frac{ReLU(p_{k})}{\sum_{k=1}^{N}ReLU(p_{k})}$$

The central server can aggregate the global model based on the optimized model weights, denoted as: $\hat{\omega }=\sum_{k=1}^{N}p_{k}\omega_{k}$. The pseudo-code for the server-side model update process is shown in Algorithm 2.
**Algorithm 2** ServerUpdate**Input:** $C_{t}$: client set; $\eta^{t}$: global learning rate;**Output:** $p_{k}^{t}$: optimized model-aggregation weights;1:Initialize $\omega_{S}^{0}$;2:**for** each client $k\in C_{t}$ in parallel **do**3:    $D_{Ck}^{syn}\leftarrow$ClientUpdate() //Receive data transmitted by the client;4:    Recover $A_{CB,k}^{t}$, $dA_{CP,k}^{t}$ and $\omega_{CB,k}^{t}$ using $D_{Ck}^{syn}$;5:    Forward propagation with $A_{CB,k}^{t}$ on $\omega_{S,k}^{t}$ and obtain the activations $A_{S,k}^{t}$;6:    Back-propagation calculate $\nabla \ell_{S}\left(\omega_{S,k}^{t}\right)$ using $dA_{CP,k}^{t}$;7:    Calculate $d_{k}^{t}$ using Equation (7) //where $\nabla F_{k}\left(\omega_{k}^{t}\right)\leftarrow \nabla \ell_{C}\left(\omega_{CP,k}^{t}\right)$;8:    Normalize $d_{k}^{t}$ using Equation (8) and collect the client dataset sizes $n_{k}$;9:    Calculate the optimized model-aggregation weights $p_{k}^{t}$ using Equation (9);10:    Model aggregation ${\hat{\omega }}_{CB}^{t}=\sum_{k=1}^{N}p_{k}^{t}\omega_{CB,k}^{t}$;11:    Generate $D_{Sk}^{syn}$ based on $A_{S,k}^{t}$, $dA_{S,k}^{t}$, and ${\hat{\omega }}_{CB}^{t}$, and send it to the clients;12:    Update the server model $\omega_{S,k}^{t+1}\leftarrow \omega_{S,k}^{t}-\eta^{t}\nabla \ell_{S}\left(\omega_{S,k}^{t}\right)$;13:**end for**

### 3.4. SplitLPF Algorithm Analysis

The pseudo-code for the training process of SplitLPF as shown in Algorithm 3.
**Algorithm 3** SplitLPF**Input:** *E*: global iteration times; *N*: number of clients;**Output:** $\omega_{CP,k}^{t}$: personalized model; $p_{k}^{t}$: optimized model-aggregation weights;1:Split the model into $\omega_{CB}$, $\omega_{S}$, $\omega_{CP}$;2:**for** t←1 to *E* **do**3:    **for** k←1 to *N* in parallel **do**4:        ClientForwordProp($\omega_{CB,k}^{t}$) // Client forward propagation;5:        ServerUpdate($\omega_{S,k}^{t}$) // Updates to the offloading model for the central server;6:        $\omega_{CP,k}^{t}\leftarrow$ClientBackProp($\omega_{CP,k}^{t-1}$) // Client-side personalized training and back propagation;7:        $p_{k}^{t}\leftarrow$ServerUpdate($dA_{CP,k}^{t}$) //Server-side back propagation with aggregation of client-side base models and update of server-side models;8:        ClientBackProp($dA_{S,k}^{t}$) //The client performs back propagation and updates the local model;9:    **end for**10:**end for**

#### 3.4.1. Complexity Analysis

In the training process of SplitLPF, each client trains the local model and sends it to the server (line 4), with a time complexity that equals o(1). The central server forward propagates to update the intermediate model (line 5), with a time complexity that equals o(N). Each client performs personalized training and back propagates it (line 6), with a time complexity that equals o(1). The central server back propagation aggregates client-side base models and updating server-side models (line 7), with a time complexity that equals o(N). The client back propagates and updates the local model (line 8), with a time complexity that also equals o(1). Thus, the total time complexity of one model update for client *k* is o(2N). The total time complexity of SplitLPF is $o(N^{2}E)$ through the cycle iteration. In terms of space complexity, SplitLPF additionally stores $w^{t}$, $D^{syn}$ and $\eta^{t}$, and they are both fixed-size parameters. Therefore, SplitLPF also has the same space complexity, o(N), with SL as well.

#### 3.4.2. Total Cost Analysis

Assume that *N* is the number of clients, *P* is the total data size, *C* is the size of the activations that pass through the model trimming layer when considering one input sample from a single client, *S* is the total synthetic data size, *R* is the rate of communication between the client and the server, *T* is the time taken for one forward and backward propagation on the full model with dataset of size *P*, $T_{1}$ is the time to generate synthetic data based on real data, $T_{agg}$ is the time required to perform the model aggregation, |W| is the the size of the full model, and β is the ratio of the full model size available to the client in SL/SplitFed, i.e., $\left|W_{C}\right|=\beta |W|$, since clients download and upload client model updates before and after training respectively. Therefore, the size of each client communication becomes 2β|W|. Based on the above assumptions, we compare the per-client communication cost, total communication cost, and total model training time in different approaches, as shown in Table 1.

### 3.5. Fairness Theory Analysis

This subsection will delve into exploring the convergence bounds of model training. Subsequently, based on this exploration, the effects of aggregation weights $p_{k}$, dataset size $n_{k}$, and local and global gradient discrepancy $d_{k}$ on convergence are obtained, and it is further concluded that dataset size $n_{k}$ and gradient direction discrepancy $d_{k}$ should be taken into account when determining the aggregation weights $p_{k}$.

Given the personalized model partitioning employed in this paper, for the sake of simplicity in analysis, we will only consider the influence of client base models during model aggregation, i.e., $\omega_{k}\leftarrow \omega_{CB}$. Assumptions such as local objective function smoothness and bounded variance of the gradient used in the literature [44,45,46,47] are first employed in order to analyze the convergence of the model.

**Theorem 1.**  *Assuming the objective function is L-smooth and the gradients have bounded variance, when we set the global learning rate $\eta <\frac{1}{L}$, the upper bound of convergence of the optimization is shown in Equation (12):*

(12) $$\begin{array}{cc} \min_{t}\;E||\nabla F\left(w^{t}\right){\left|\right|}^{2} & \leq \frac{2}{1-4(H+2K)}[\frac{1}{\eta T}\left(F\left(w^{0}\right)-F_{\inf}\right)\\ \; & +2HB+\frac{2AH}{N}\sum_{k=1}^{N}d_{k}+K\left(\sigma^{2}+4\left(A\sum_{k=1}^{N}p_{k}d_{k}+B\right)\right)] \end{array}$$

*where $H=N\sum_{k=1}^{N}{\left(n_{k}-p_{k}\right)}^{2}$, $K=\frac{L^{2}}{\gamma }\sum_{\tau =0}^{\gamma }\frac{2\eta^{2}\tau }{\left(1-4\eta^{2}L^{2}\tau \right)}$, A and B are both positive constants, and $F_{\inf}$ and $\sigma^{2}$ correspond to the bounded scalar and variance of gradients, respectively.*

A detailed proof is provided in Appendix A.

Considering stricter boundary conditions typically corresponds to superior optimization outcomes. Hence, this paper delves into exploring the impact of the parameter aggregation weight $p_{k}$ on the upper-bound conditions. From Theorem 1, it is apparent that the weight $p_{k}$ is primarily correlated with *H*, which corresponds to the three components in the convergence bounds. To shrink the bounds, consider optimizing $p_{k}$ to minimize the upper bound. As a result, a concise expression for the relationship between the aggregation weight $p_{k}$, the dataset size $n_{k}$, and the discrepancy $d_{k}$ between the local and global gradient can be derived.

**Lemma 1.**  *Assuming η<1/L, for more stringent upper bounds, the aggregation weight $p_{k}$ can be characterized as shown in Equation (13):*

(13) $$p_{k}\propto n_{k}+Jd_{k}$$

*where J is a positive constant.*

Detailed proofs can be found in Appendix B.

In stricter upper limits, the aggregation weight $p_{k}$ correlates with the dataset size $n_{k}$ and the discrepancy $d_{k}$ between local and global gradients. For resource-constrained devices, the dataset size is typically relatively smaller. Therefore, further adjustments in the contributions of $n_{k}$ and $d_{k}$ can lead to the optimal model weight. Optimizing Lemma 1 appropriately results in the expression for the aggregation weight in this paper, i.e., $p_{k}=\alpha n_{k}+\left(1-\alpha \right)d_{k}$, as shown in Equation (10). Thus, fine-tuning the hyperparameters α provides an optimal selection for model performance.

## 4. Experiment and Result Analysis

**Figure 3:**
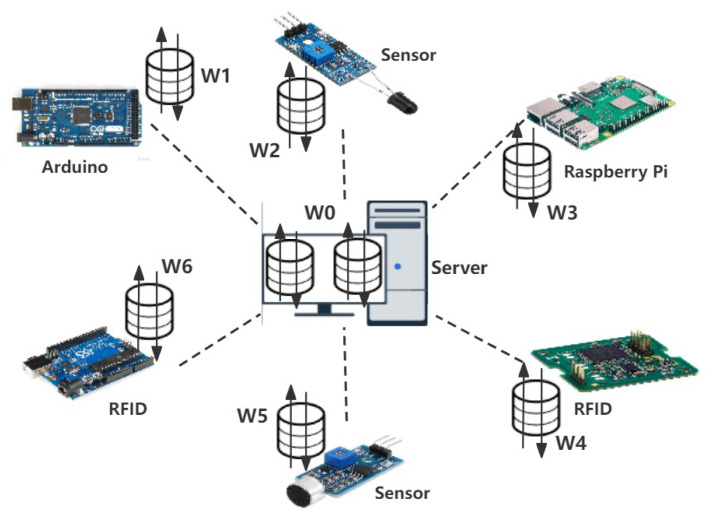
Experimental scenario setup in IoT simulated environment.

The performance of SplitLPF in IoT environments was evaluated through experiments. Consider a typical IoT application scenario as shown in Figure 3. In general, IoT scenarios usually contain both strong devices (resource-rich clients) and weak devices (resource-constrained clients). Weak devices typically possess limited computational and storage resources, such as sensors and RFID tag cards. In contrast, strong devices have more computational and storage capabilities, capable of handling more complex tasks and algorithms, such as NVIDIA Jetson Nano and cloud servers. The experiments simulated a multi-machine deployment to adapt to the distributed environment requirements of split learning. To better manage resources and reduce unnecessary waste, a configuration comprising 10 commonly used IoT devices (including four Raspberry Pi, four STM32 boards, and two NVIDIA Jetson Nano) and one central server was chosen. In this configuration, the scaled-down Raspberry Pi and STM32 boards emulated weak devices, while the NVIDIA Jetson Nano emulated a strong device. In the experiment, the hardware environment of IoT devices encompassed CPU Cortex-A53, Cortex-M0, and Cortex-A57 ARM, with RAM capacities of 1G and 2G. Operating systems included Raspbian GNU/Linux10 (buster), FreeRTOS, and Ubuntu 18, with Python 3.7 as the development language. The central server (a laptop) featured an Intel i5-13500HQ CPU, 32GB RAM, 1TB ROM, and GPU RTX4060, and it ran on Windows 11. Software environment comprised Pycharm as the development environment and Python 3.9 as the programming language. In order to validate the effectiveness of SplitLPF, this paper conducts experiments on Fashion-MNIST (FMNIST), Extended MNIST (EMNIST), and CIFAR-10 datasets, all of which use the PyTorch framework for training deep learning models. These experiments aim to demonstrate the performance of SplitLPF under different datasets.

### 4.1. Datasets

**Figure 4:**
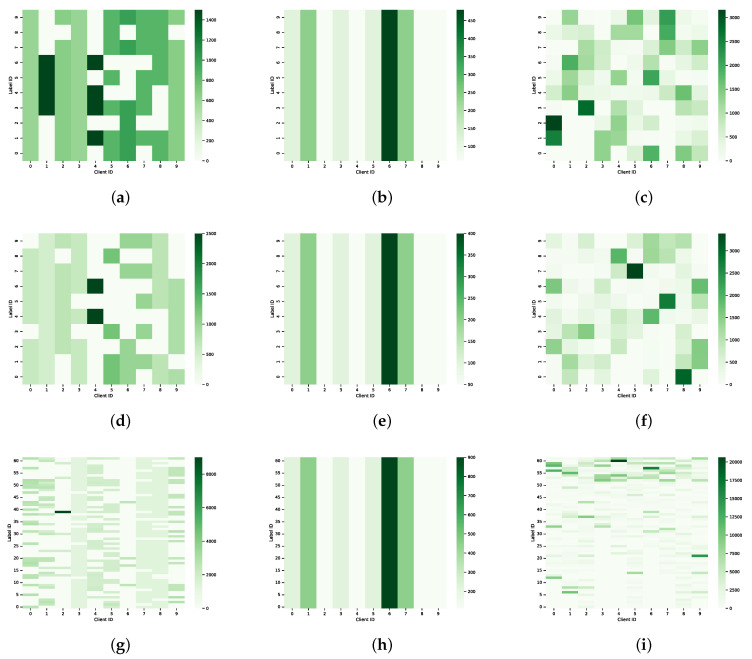
Three non-IID forms of FMNIST, CIFAR-10, and EMNIST; (**a**) FMNIST UDD; (**b**) FMNIST UDS; (**c**) FMNIST UDDS; (**d**) CIFAR-10 UDD; (**e**) CIFAR-10 UDS; (**f**) CIFAR-10 UDDS; (**g**) EMNST UDD; (**h**) EMNST UDS; (**i**) EMNST UDDS.

The experiment evaluated the proposed framework using three popular datasets. FMNIST and CIFAR-10 datasets contain images from 10 categories each, while EMNIST, an extension of MNIST, includes 62 categories. All three datasets were divided into a training set and a test set using a standard data splitting approach. To simulate data heterogeneity seen in real-world scenarios, we used three forms of non-IID data segmentation for each dataset, as shown in Figure 4. Unbalanced Data Distribution (UDD): This scenario simulated uneven data distribution by altering the number of label categories within client datasets. In this case, a different number of labeling categories are distributed among different clients, i.e., weak clients usually have fewer labeling categories than strong clients but not less than one. For instance, among 10 clients, different subsets of label categories were allocated to each client, as illustrated in Figure 4a,d,g. Unbalanced Data Size (UDS): Data size was allocated to clients based on a power-law rule, creating a scenario where some clients had fewer data sizes while others had more. All clients possessed all label categories, but weak clients typically had less data than strong clients. For example, for the CIFAR-10 dataset with 10 clients, 500, 1000, 2000, and 4000 data are assigned to 4, 3, 2, and 1 clients, as shown in Figure 4b,e,h. Unbalanced Data Distribution and Size (UDDS): We use the Dirichlet distribution [48] ${Dir}_{N}\left(\beta \right)$ for data partitioning, where *N* is the number of clients and β determines the degree of non-independent homogeneous distribution, i.e., the smaller the value of β, the more unbalanced the data distribution. In this way, the scenario of uneven data distribution and data size between clients is simulated. For example, the ${Dir}_{10}\left(0.5\right)$ distribution for 10 clients is shown in Figure 4c,f,i. Across these simulated heterogeneous data scenarios, the weak client is significantly weaker than the strong client in terms of both data distribution and data size, and these simulated scenarios mimic, as much as possible, the different data heterogeneity scenarios that may occur in real IoT environments.

### 4.2. Baseline and Experimental Setup

The paper compares SplitLPF with state-of-the-art methods focused on personalization and fairness, including FedPer [49], which employs an architecture-based personalized federated learning method combining base and personalized layers; FedPAC [50], which achieves personalized federated learning through feature alignment and collaborative classifiers; FedCI [51], which employs client evaluation and uses evaluation outcomes as fairness-aware aggregation weights in federated learning; Ditto [35], an expandable federated multi-task learning framework that achieves fair federated learning through personalization; SplitGP [30], a personalized splitting learning method capable of capturing both generalization and personalization functionalities; and PFSL [29], a personalized fair splitting learning approach that combines transfer learning with lightweight personalization and cooperative fairness. Additionally, the comparison includes the benchmark federated learning method, FedAvg, and the federated splitting learning method, SplitFed [13]. For the model settings, a convolutional neural network (CNN) consisting of three convolutional layers and three fully connected layers was employed to process the FMNIST and EMNIST datasets. For the CIFAR-10 dataset, a ResNet18 neural network with a depth of 18 was used. In the split learning scenario, we offload most of the computational tasks of the model to the central server, and the number of front, middle, and back layers in the model structure is allocated based on the computational latency and computational complexity. Among them, the back part of the model retains the last layer of full connectivity with dropout operation to avoid model inference. SGD was utilized for model optimization during training, with an initial global learning rate set to η = 0.05 and a momentum change rate of 0.9. In terms of parameter settings, the training batch size was set to *B* = 128, the local iterations for all methods were set to *e* = 2, and the global iterations were set to *E* = 100. The default weight coefficient was set to α = 0.5, and the non-identical distribution degree β was set to 0.5. In the personalization settings, the local learning rate $\eta_{c}$ was dynamically adjusted based on the model accuracy achieved by each client during training.

### 4.3. Evaluation Index

To compare the performance of SplitLPF with other methods in terms of personalization and fairness, this paper employed a variety of evaluation metrics, including Test Accuracy and Personalized Accuracy: this metric is used to assess the model’s performance by comparing the test accuracy achieved during model training; Scaled Pearson Correlation (PC) [52]: this metric is used to compare the test accuracy φ obtained when clients train independently with the test accuracy θ obtained when they collaborate through fairness mechanisms in FL, and it is calculated as $PC=\frac{Cov(\varphi ,\theta )}{\sigma_{\varphi }\sigma_{\theta }}$, where Cov represents covariance and σ represents standard deviation; and Jain’s Fairness Index (JFI) [53]: this metric is used to compare the accuracy distributions of *N* clients on a local dataset and combine their accuracy scores into a vector *s*, which is denoted as $JFI=\frac{{\left(\sum_{i=1}^{N}s_{i}\right)}^{2}}{N\sum_{i=1}^{N}s_{i}^{2}}$. In the literature [52,53], PC and JFI are used to assess method fairness. In addition, this paper calculates the standard deviation of test performance between clients according to Definition 1 to further measure fairness.

### 4.4. Analysis of Experimental Results

To validate the performance of SplitLPF, a comparison was made against other state-of-the-art methods across three datasets. The average test accuracy of model training under three distinct data heterogeneity scenarios is shown in Table 2.

From the Table 2, it is easy to see that SplitLPF presents satisfactory performance in terms of test accuracy for different datasets in all three cases of data heterogeneity. Notably, in all three datasets, adopting the UDDS scenario with Dirichlet distribution for data partitioning showcased higher test accuracy compared to focusing solely on uneven data distribution in UDD scenarios or differing data sizes in UDS scenarios. The reason for this is that the data distribution and the number of data in the UDDS scenario are appropriately compensated for each client, so that each client has access to a certain number of data, allowing the model to learn and adapt to different data features in a more comprehensive way, thus improving the accuracy of the model on the test set. In addition, in Table 2, the average test accuracy of SplitLPF exceeds that of the benchmark federated learning method FedAvg, and for the benchmark method SplitFed with the same splitting strategy, the test accuracy of SplitLPF is significantly higher than that of this benchmark as well. This indicates the superior performance of SplitLPF in the experimental evaluation. The enhanced performance of SplitLPF might stem from its utilization of optimized methods for aggregating model weights, which effectively integrate pertinent information from client devices, resulting in improved global model accuracy. In addition, consider that in the model design, SplitFed will make the accuracy rate somewhat lower because it needs noise to the uploaded gradients and labels to protect the privacy leakage during the model-training process.

Compared to other state-of-the-art methods, such as the FedPer, FedPAC, and SplitGP methods for personalized settings, it can be seen from Table 2 that SplitLPF all performs slightly higher than other personalized schemes. For instance, in the UDS scenario of the FMNIST dataset, SplitLPF demonstrates average test accuracy improvements of 0.98%, 0.33%, and 0.82% for FedPer, FedPAC, and SplitGP, respectively. Therefore, compared with existing methods, SplitLPF shows good performance under different datasets and data heterogeneity conditions. A horizontal comparison among existing personalized methods from Table 2 reveals that both FedPAC and SplitGP achieve higher average test accuracy than FedPer. This is primarily attributed to FedPAC employing effective feature alignment strategies and a robust classifier collaboration mechanism, which leads to a more consistent feature space among different clients, facilitating better collaboration and integration of information from various client sources. Meanwhile, SplitGP leverages controllable parameters to adjust the boundaries between personalization and generalization, effectively promoting collaborative training among clients. In contrast, while FedPer’s personalized layer offers a certain degree of client customization, its integration capability might be slightly lacking, resulting in a slightly lower average test accuracy compared to FedPAC and SplitGP methods. PFSL, a split learning approach that considers both personalization and fairness, demonstrates an average test accuracy close to that of SplitLPF and surpasses other baseline methods. This can be attributed to PFSL’s utilization of transfer learning strategies and freezing weights to achieve client personalization effectively, enabling it to adapt efficiently to various data heterogeneity scenarios.

**Figure 5:**
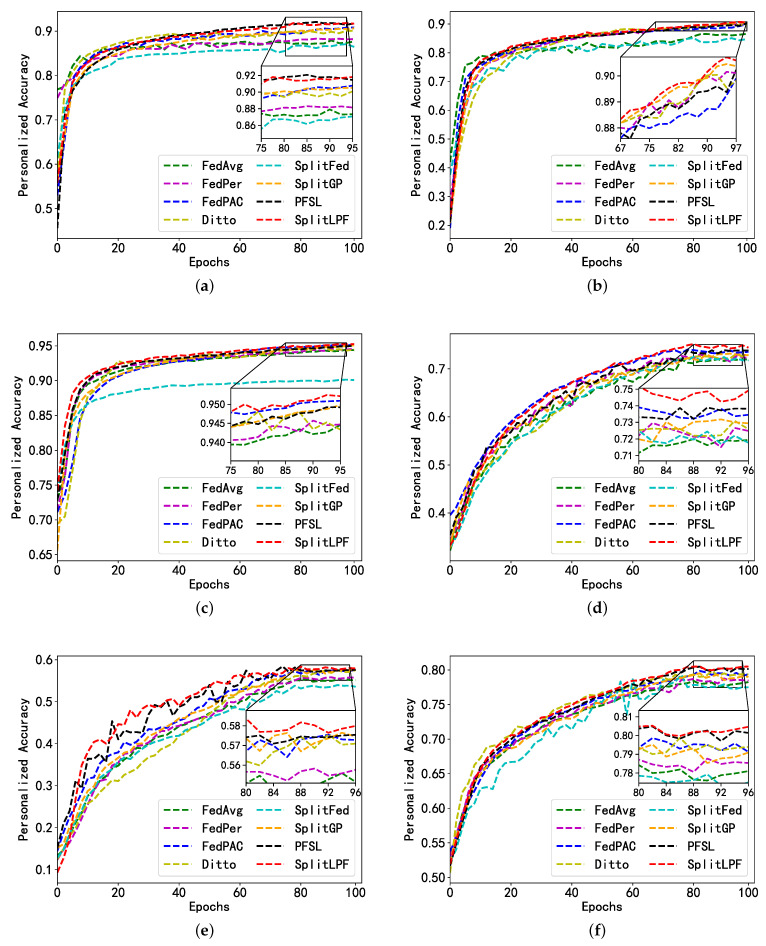
Personalized accuracy of FMNIST and CIFAR10 under different non-IID data; (**a**) FMNIST UDD; (**b**) FMNIST UDS; (**c**) FMNIST UDDS; (**d**) CIFAR-10 UDD; (**e**) CIFAR-10 UDS; (**f**) CIFAR-10 UDDS.

In order to better demonstrate the effectiveness of personalization in SplitLPF, all the personalization methods as well as the benchmark methods were simulated and compared with SplitLPF. The experimental performance was evaluated on FMNIST and CIFAR-10 datasets, and the experimental results are shown in Figure 5. The experimental results are depicted in Figure 5. It is evident from Figure 5 that SplitLPF outperforms other methods in terms of personalized accuracy, while benchmark methods show relatively lower accuracy in comparison to personalized methods. Among the personalized methods considered, two split learning methods, PFSL and SplitGP, were assessed. Both adopted a model partitioning strategy that aimed to adapt better to resource-constrained devices. It can be observed from Figure 5 that both the methods perform well in terms of performance. PFSL leverages transfer learning, enabling the model to utilize abundant source domain data to assist in personalized training on resource-constrained weaker clients, thereby enhancing the model’s generalization ability. Simultaneously, model partitioning allows personalized training to focus more on specific tasks and data features at the client’s local level, effectively boosting accuracy. Meanwhile, SplitGP, through parameter control, adjusts the degree of personalization, enabling it to adapt to local data features on clients while maintaining generalization over global data. This balance allows the model to better cater to the requirements of different clients, consequently enhancing accuracy.

In order to validate the fairness achieved by SplitLPF through optimized model-aggregation weights, Table 3 presents the comparative results between SplitLPF and other baseline methods in terms of fairness metrics. Scaled Pearson Correlation (PC), Jain’s Fairness Index (JFI), and standard deviation (Std) are employed in Table 3 as indicators measuring fairness. Higher PC and JFI values alongside lower Std typically indicate a more balanced and fair distribution of benefits within the evaluation. Observing Table 3, it can be noted that SplitLPF achieves higher levels of fairness compared to other methods. Specifically, on the FMNIST and EMNIST datasets, SplitLPF achieves a PC (%) of over 90, indicating strong correlation. Moreover, the JFI (%) on all datasets exceeds 98. In addition, the standard deviation of SplitLPF on the EMNIST and CIFAR-10 datasets is kept at a low take. This means that the performance gap between different clients is small, and both achieve good performance.

In fairness-driven distributed learning approaches such as FedCI, Ditto, and PFSL, it is apparent from Table 3 that they show better performance than methods that focus on personalization. The reason behind this phenomenon likely lies in the different strategies adopted by these methods. In FedCI, fairness is achieved by fostering collaboration and information sharing, ensuring that each participant obtains reasonable gains in federated learning, thus enhancing overall fairness. Ditto achieves a higher level of fairness through a personalization mechanism and a reasonable distribution of proceeds. In contrast, PFSL maintains not only high levels of personalized accuracy but also superior fairness. This illustrates that in method design, personalization and fairness can be compatible. Taking inspiration from this, the present study optimizes both personalized models and fairness methods, aiming to better satisfy the dual requirements of personalization and fairness.

**Figure 6:**
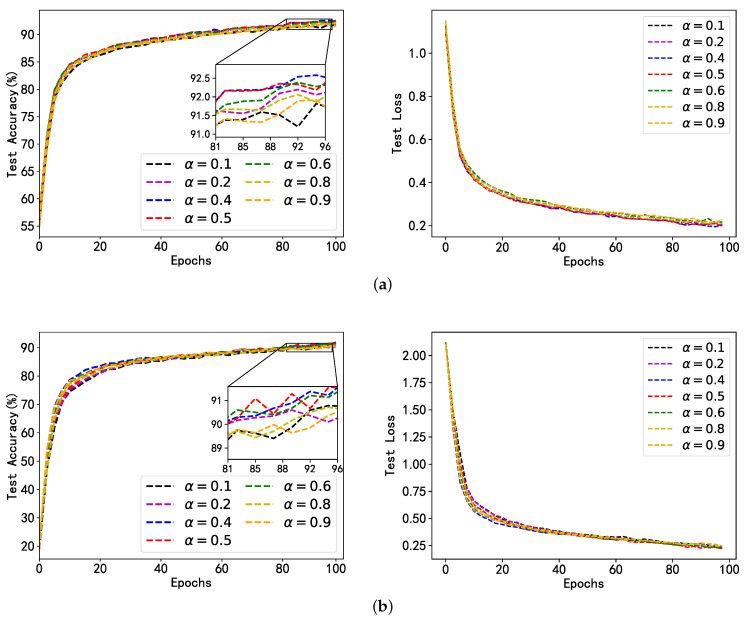
Model accuracy and loss of SplitLPF under different α conditions (based on FMNIST dataset); (**a**) Performance of FMNIST under UDD; (**b**) Performance of FMNIST under UDS.

**Figure 7:**
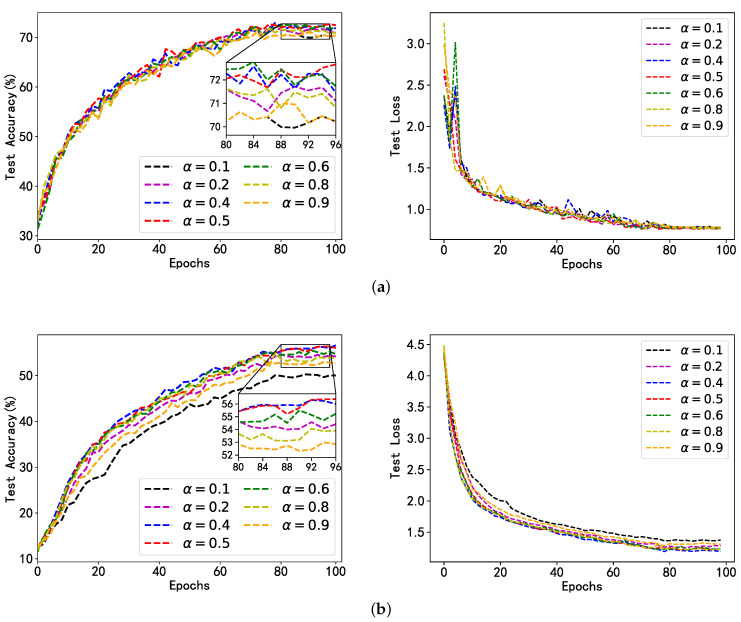
Model accuracy and loss of SplitLPF under different α conditions (based on CIFAR-10 dataset); (**a**) Performance of CIFAR-10 under UDD; (**b**) Performance of CIFAR-10 under UDS.

In the fairness mechanism, the contribution weights of dataset size and gradient direction disparity to model performance are adjusted by tuning the hyperparameter α. Different values of the hyperparameter α will be attempted to assess the impact of the contribution ratio of the dataset size and gradient direction disparity on SplitLPF’s performance. The experimental results are presented in Figure 6 and Figure 7. We can see that Figure 6 evaluates the impact of hyperparameters α on the experimental results on the FMNIST dataset, and Figure 7 evaluates the impact of hyperparameters on the experimental results on the CIFAR-10 dataset. We controlled the value of the hyperparameter a between [0–1], specifically taking values of 0.1, 0.2, 0.4, 0.5, 0.6, 0.8, and 0.9. For each of these values, 100 global iterations and two local iterations are conducted.

From the results in Figure 6 and Figure 7, it can be observed that with different data heterogeneity and different datasets, SplitLPF’s performance is significantly better when α is set to 0.4, 0.5, and 0.6 compared to when α is set to 0.1, 0.2, 0.8, and 0.9. Additionally, the performance is slightly lower when α is set to 0.1 and 0.9 compared to other values. This indicates that relying solely on either local dataset size or the disparity between gradient direction and the global gradient is not the optimal choice. Only by properly allocating the weights for both factors and ensuring an appropriate contribution ratio between them, it is more likely to obtain the best model weights and provide the optimal selection for model performance. Based on this, we optimize the model performance by adjusting the value of the hyperparameter α in order to better optimize the model performance.

**Figure 8:**
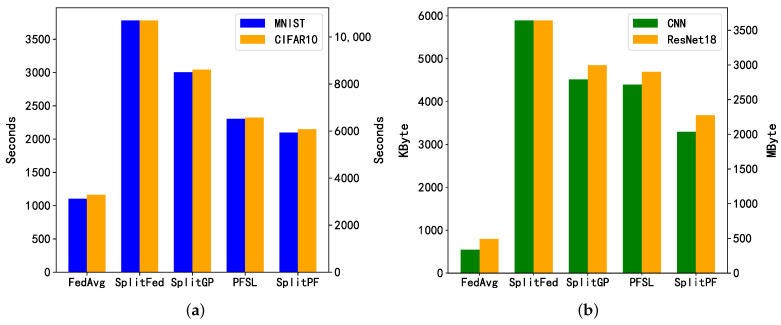
Comparison of time overhead and communication overhead between different methods; (**a**) Time overhead; (**b**) Communication overhead.

In order to evaluate the resource consumption of SplitLPF, we compared several split learning methods that introduce certain communication overheads due to model partitioning. These include SplitFed, SplitGP, and PSFL, as well as the benchmark federated learning method FedAvg. Figure 8 illustrates the comparison of these methods in terms of time and communication overhead. From Table 1, it is evident that the communication in the federated learning approach mainly comes from downloading and uploading models, while the data communication in the split learning approach consists of transmitting intermediate results and synchronizing client models. Therefore, the communication cost incurred by FedAvg is significantly lower than that of SplitFed, SplitGP, PSFL, and SplitLPF.

In Figure 8b, the disparity in communication overhead resulting from training different models can be observed. The vertical coordinates on the left side of the figure represent the criteria for the communication overhead of different methods when training on the CNN model, and the vertical coordinates on the right side represent the criteria for the communication overhead of different methods when training on the ResNet18 model. Although SplitLPF incurs higher communication costs due to model partitioning, it employs a lightweight synthetic data transfer approach. This method utilizes smaller batch data to learn representative information from large-scale data effectively and can recover data information efficiently, thereby reducing some communication load. Observing Figure 8b, it is evident that compared to the existing personalized fair SL method PSFL, SplitLPF’s communication overhead is reduced by approximately 24%.

In Figure 8a, the comparison of time expenditures for training over 20 rounds across different methods is displayed. The power consumption of IoT devices intensifies as the training time increases, and some resource-constrained devices may power down midway due to load. Therefore, the selected training rounds were optimized to be the appropriate choice. PFSL and SplitLPF exhibit reduced time expenditures compared to other split learning methods. This is largely attributed to the fact that they employ the parallel training of clients and that the servers effectively support the parallel processing of client data. Moreover, SplitLPF’s time expenditure is slightly lower than PFSL, possibly due to the increased efficiency resulting from reduced communication data volume. In summary, compared to other split learning methodologies, SplitLPF demonstrates higher efficiency and lower communication costs in terms of resource consumption.

### 4.5. Extended Discussion

#### Privacy Analysis

In the proposed SplitLPF framework, each client undergoes specific personalized design to better adapt to the characteristics of different devices, enhancing model performance and adaptability. The central server (referred to as the third party) only serves the role of executing offloading computational tasks to participate in model training, without delving into the detailed client-side operations or accessing information about their data labels. Furthermore, when updating the client’s base model, a separate global learning rate is used for model updates. In contrast, the update of the client personalized model is controlled by the local learning rate. Clients locally store data labels and dynamic local learning rates. Thus, unlike traditional two-phase split learning, in this framework, the server lacks access to client data labels, significantly increasing the difficulty of model inversion attacks. In addition, unlike the SplitFed learning approach, SplitLPF does not require data to be noised during data transmission. Instead, lightweight synthetic data is used for transmission, effectively reducing the risk of data leakage. In specific scenarios, a public-private key encryption mechanism can be employed to further enhance data confidentiality, although this might lead to a slight increase in communication overhead.

## 5. Conclusions

In this paper, we propose SplitLPF, a personalized and fair split learning framework for resource-constrained IoT devices, which aims to address the difficulty of resource-constrained clients in traditional federated learning to efficiently train complex local models, as well as to deal with the challenges posed by the heterogeneity of data and the uneven distribution of benefits in IoT environments. In the IIoT domain, data heterogeneity arises from the diversity of sensing capabilities, environmental conditions, and data characteristics of different devices. Traditional SL training methods may lead to performance degradation in such a heterogeneous environment. On the other hand, benefit imbalance manifests as some smaller devices contributing significantly but not receiving proportional benefits during model training. This could lead to their withdrawal from cooperation, endangering the entire learning process. The SplitLPF framework achieves improved adaptation to specific application scenarios for resource-constrained IoT devices through model splitting, flexible task allocation, and support for personalized training. In terms of model aggregation, an optimized model weight aggregation method is employed to ensure fair benefit distribution by reasonably allocating the contribution proportion, thereby providing the best model performance selection, and to achieve collaborative fairness. This promotes a more balanced and sustainable collaboration. The experimental results demonstrate that the SplitLPF framework achieves higher accuracy through personalized training methods, even in scenarios with diverse data heterogeneity. Furthermore, the framework ensures collaboration fairness, providing a more stable foundation for cooperation among IoT devices. This research offers a novel approach to address distributed learning issues in the IoT, with the potential for significant breakthroughs in IoT applications.

However, the study also has limitations. For instance, SplitLPF still incurs certain communication overheads, particularly during the model segmentation and aggregation phases, thereby increasing the communication burden. Additionally, the SplitLPF method restricts the amount of shared information among different clients. This limitation might hinder the model from fully utilizing the data features of all clients, potentially reducing the model’s generalization ability. Therefore, the next step involves further addressing the communication overhead issues posed by SplitLPF. We plan to consider integrating more efficient communication technologies, such as the FedD3 framework [54], which distills instances through the dataset, requires only one-time communication, and greatly reduces communication costs. By introducing such efficient communication schemes, we aim to effectively tackle the communication cost issue while enhancing the efficiency and performance of distributed learning models, making SplitLPF adaptable to more different scenarios. In future work, we also plan to enhance the generalization ability of the model by improving the information exchange strategy. This improvement will be achieved by augmenting non-private portions of the model parameters, feature summaries, or gradient information. By doing so, the model can share partial information more extensively, fostering increased communication and information sharing among the involved parties.

## Figures and Tables

**Table 1:** Total cost analysis of the different approaches for one global epoch.

Methods	Communication Cost Per Client	Total Communication Cost	Total Model Training Time
FL	2|W|	2N|W|	$T+\frac{2|W|}{R}+T_{agg}$
SL	$\frac{2PC}{N}+2\beta \left|W\right|$	2PC+2βN|W|	$T+\frac{2\beta N|W|}{R}+\frac{2PC}{R}$
SplitFed	$\frac{2PC}{N}+2\beta \left|W\right|$	2PC+2βN|W|	$T+\frac{2\beta |W|}{R}+\frac{2PC}{NR}+T_{agg}$
SplitLPF	$\frac{4S}{N}$	4S	$T+2T_{1}+\frac{4S}{NR}+T_{agg}$

**Table 2:** Average test accuracy (%) of each method with different non-IID data.

Datasets	FMNIST			EMNIST			CIFAR-10		
Method	UDD	UDS	UDDS	UDD	UDS	UDDS	UDD	UDS	UDDS
FedAvg	86.14	80.69	91.71	75.43	69.50	83.47	61.40	40.82	71.56
FedPer	86.19	81.98	91.72	76.12	70.24	82.92	61.97	40.94	71.72
FedPAC	86.78	82.37	92.14	76.38	70.58	85.64	62.73	43.77	72.16
FedCI	86.96	82.22	92.09	76.39	70.79	85.68	62.05	43.11	72.12
Ditto	86.70	81.28	91.60	75.82	70.23	85.48	61.76	42.92	72.11
SplitFed	85.67	78.77	88.44	67.63	66.23	81.52	59.61	40.02	70.84
SplitGP	86.30	82.13	91.93	76.23	70.72	85.09	61.66	42.59	71.67
PFSL	87.40	82.43	92.32	76.43	70.98	86.14	62.22	46.14	72.48
SplitLPF	87.28	82.95	92.85	76.88	71.86	87.15	62.96	46.97	72.88

**Table 3:** The fairness index of each method is compared under different datasets.

Datasets	FMNIST			EMNIST			CIFAR-10		
Method	PC	JFI	Std	PC	JFI	Std	PC	JFI	Std
FedAvg	85.15	93.92	17.47	87.69	97.54	7.91	73.87	93.89	6.47
FedPer	85.94	94.47	17.02	90.53	97.73	7.88	73.57	94.93	6.30
FedPAC	85.31	94.05	16.85	89.81	97.20	6.74	73.43	93.37	6.19
FedCI	88.35	95.20	16.15	92.07	97.72	5.24	75.20	95.18	4.66
Ditto	87.98	95.49	16.46	91.56	97.95	6.20	74.54	96.88	4.98
SplitFed	72.72	90.96	20.36	55.44	95.00	33.19	69.64	89.79	8.37
SplitGP	78.74	91.55	17.55	77.46	96.15	15.15	72.59	92.96	7.33
PFSL	89.43	96.16	15.03	94.51	97.96	3.06	76.45	95.45	3.27
SplitLPF	91.69	98.76	14.73	91.21	98.32	2.64	78.57	98.39	3.01

## Data Availability

The fashion-mnist dataset can be downloaded at https://github.com/zalandoresearch/fashion-mnist/ (accessed on 15 July 2023), the cifar10 dataset can be downloaded at https://www.cs.toronto.edu/kriz/cifar.html (accessed on 1 June 2023), and the emnist dataset can be downloaded at https://www.itl.nist.gov/iaui/vip/cslinks/EMNIST/ (accessed on 15 July 2023).

## Abbreviations

The following abbreviations are used in this manuscript:

IoT	Internet of Things
IIoT	Industrial Internet of Things
FL	Federated learning
SL	Split learning
SplitLPF	Split Learning with Personalized Fairness
PSL	Parallel split learning
IID	Identically and independently distributed
Non-IID	Non-independent and identically distributed
SGD	Stochastic gradient descent
FMNIST	Fashion-MNIST
EMNIST	Extended MNIST
UDD	Unbalanced Data Distributed
UDS	Unbalanced Data Size
UDDS	Unbalanced Data Distributed and Size
PC	Pearson Correlation
JFI	Jain’s Fairness Index
Std	Standard deviation
CNN	Convolutional neural network

## Appendix A

Considering the simplified model training objective in this paper, the convergence proof is provided below. First, the global objective function before optimization is defined as $\nabla F\left(\omega \right)=\sum_{k=1}^{N}n_{k}\nabla F_{k}\left(\omega \right)$, where $\sum_{k=1}^{N}n_{k}=1$. Let $G_{k}$ be the local accumulated stochastic gradients, represented as $G_{k}=\frac{1}{\gamma }\sum_{\tau =1}^{\gamma }\nabla F_{k}\left(\omega_{k}^{\left(t,\tau \right)}\right)$. For each round, the global model update can be described as: $w^{t+1}-w^{t}=\eta \sum_{k=1}^{N}p_{k}G_{k}$, where $\sum_{k=1}^{N}p_{k}=1$. This analysis relies on assumptions related to local objective function smoothness and bounded gradient variance, as found in the literature [44,45,46,47].

**Proposition A1.** 
*(Smoothness). For each local objective function that is L-Lipschitz smooth, it has for all k∈[1,N]: $||\nabla F\left(\omega_{k+1}\right)-\nabla F\left(\omega_{k}\right)\left|\right|\leq L||\omega_{k+1}-\omega_{k}\left|\right|$, its equivalence condition is:*

(A1)
$$\nabla F\left(\omega_{k+1}\right)-\nabla F\left(\omega_{k}\right)\leq -\langle \nabla F\left(\omega_{k}\right),\omega_{k+1}-\omega_{k}\rangle +\frac{L}{2}\left|\right|\omega_{k+1}-\omega_{k}{\left|\right|}^{2}$$

**Proposition A2.** 
*(Bounded gradient and bounded variance). For each client i, the local stochastic gradient is unbiased, i.e., $E\left[G_{k}\left(x;\omega_{k}\right)\right]=\nabla F_{k}\left(\omega \right)$. Moreover, it possesses a bounded variance, i.e., $E\left[G_{k}\left(x;\omega_{k}\right)-\nabla F_{k}\left(\omega \right)\right]\leq \sigma^{2}$.*

**Proposition A3.** 
*(Bounded discrepancy and bounded scalar). For each local objective function $\nabla F_{k}\left(\omega \right)$, constants A and B greater than zero exist, such that: $|\nabla F_{k}{\left(\omega \right)-\nabla F\left(\omega \right)|}^{2}\leq Ad_{k}+B$, where $d_{k}$ represents the local-global discrepancy. The global objective function is bounded by $F_{\inf}$.*

**Proposition A4.** *(Conditional expectation). For a sequence of random matrices ${\left\{M_{t}\right\}}_{t}^{T}$ satisfying the zero conditional expectation $E[M_{t}|M_{t-1},M_{t-2},...,M_{1}]=0$ and the independence assumption $P\left[M_{t}|M_{t-1},M_{t-2},...,M_{1}\right]=P\left[M_{t}\right]$, there is $E[{\left||\sum_{t=1}^{T}M_{t}|\right|}^{2}]=\sum_{t=1}^{T}E\left[{\left||M_{t}|\right|}^{2}\right]$*.

**Proof of Theorem A1.** Next, we will present the derivation of the convergence process. Given that the global gradient is smooth, based on proposition (A1), we can obtain:

(A2) $$\begin{array}{cc} F\left(w^{t+1}\right)-F\left(w^{t}\right) & \leq -\langle \nabla F\left(w^{t}\right),\left(w^{t+1}-w^{t}\right)\rangle +\frac{L}{2}\left|\right|w^{t+1}-w^{t}{\left|\right|}^{2}\\ \; & =-\eta \underset{C_{1}}{\underset{︸}{\langle \nabla F\left(w^{t}\right),\sum_{k=1}^{N}p_{k}G_{k}\rangle }}+\frac{L\eta^{2}}{2}\left|\right|\sum_{k=1}^{N}p_{k}G_{k}{\left|\right|}^{2} \end{array}$$

According to ${\left||a|\right|}^{2}+{\left||b|\right|}^{2}-||a-{b||}^{2}-2\langle a,b\rangle =0$, $C_{1}$ is further expressed as:

(A3) $$\begin{array}{cc} C_{1}= & \langle \nabla F\left(w^{t}\right),\sum_{k=1}^{N}p_{k}G_{k}\rangle =\frac{1}{2}||\nabla F\left(w^{t}\right){\left|\right|}^{2}\\ & +\frac{1}{2}\left|\right|\sum_{k=1}^{N}p_{k}G_{k}{\left|\right|}^{2}-\frac{1}{2}\underset{C_{2}}{\underset{︸}{||\nabla F\left(w^{t}\right)-\sum_{k=1}^{N}p_{k}G_{k}{\left|\right|}^{2}}} \end{array}$$

$C_{2}$ can be expressed as:

(A4) $$\begin{array}{cc} C_{2}= & ||\nabla F\left(w^{t}\right)-\sum_{k=1}^{N}p_{k}G_{k}{\left|\right|}^{2}\\ \; & =\left|\right|\sum_{k=1}^{N}\left(n_{k}-p_{k}\right)\nabla F_{k}\left(w^{t}\right)+\sum_{k=1}^{N}p_{k}\left(\nabla F_{k}\left(w^{t}\right)-G_{k}\right){\left|\right|}^{2} \end{array}$$

According to $||a+{b||}^{2}\leq {2||a||}^{2}+{2||b||}^{2}$ and Cauchy’s inequality, $C_{2}$ is further expressed as:

(A5) $$\begin{array}{cc} C_{2} & \leq 2||\sum_{k=1}^{N}\left(n_{k}-p_{k}\right)\nabla F_{k}\left(w^{t}\right){\left|\right|}^{2}+2||\sum_{k=1}^{N}p_{k}\left(\nabla F_{k}\left(w^{t}\right)-G_{k}\right){\left|\right|}^{2}\\ \; & \leq 2\left[\sum_{k=1}^{N}\left|\right|n_{k}-p_{k}{\left|\right|}^{2}\right]\underset{C_{3}}{\underset{︸}{\left[\sum_{k=1}^{N}{||\nabla F_{k}\left(w^{t}\right)\left|\right|}^{2}\right]}}\\ \; & +2\sum_{k=1}^{N}p_{k}\underset{C_{4}}{\underset{︸}{||\nabla F_{k}\left(w^{t}\right)-G_{k}{\left|\right|}^{2}}} \end{array}$$

According to $||a+{b||}^{2}\leq {2||a||}^{2}+{2||b||}^{2}$, $C_{3}$ can be expressed as:

(A6) $$\begin{array}{cc} C_{3}= & \sum_{k=1}^{N}||\nabla F_{k}\left(w^{t}\right){\left|\right|}^{2}=\sum_{k=1}^{N}\left|\right|\left(\nabla F_{k}\left(w^{t}\right)-\nabla F\left(w^{t}\right)\right)+\nabla F\left(w^{t}\right){\left|\right|}^{2}\\ \; & \leq 2\sum_{k=1}^{N}||\nabla F_{k}\left(w^{t}\right)-\nabla F\left(w^{t}\right){\left|\right|}^{2}+2\sum_{k=1}^{N}||\nabla F\left(w^{t}\right){\left|\right|}^{2} \end{array}$$

According to proposition (A3), $C_{3}$ is further expressed as:

(A7) $$\begin{array}{cc} C_{3} & \leq 2\sum_{k=1}^{N}\left(Ad_{k}+B\right)+2\sum_{k=1}^{N}||\nabla F\left(w^{t}\right){\left|\right|}^{2}\\ \; & \leq 2N(||\nabla F\left(w^{t}\right){\left|\right|}^{2}+B)+2A\sum_{k=1}^{N}d_{k} \end{array}$$

In $C_{4}$, according to proposition (A4), $C_{4}$ can be expressed as:

(A8) $$\begin{array}{cc} C_{4}= & ||\nabla F_{k}\left(w^{t}\right)-G_{k}{\left|\right|}^{2}=||\nabla F_{k}\left(w^{t}\right)-\frac{1}{\gamma }\sum_{\tau =0}^{\gamma }\nabla F_{k}\left(w^{\left(t,\tau \right)}\right){\left|\right|}^{2}\\ \; & =\left|\right|\frac{1}{\gamma }\sum_{\tau =0}^{\gamma }(\nabla F_{k}\left(w^{t}\right)-\nabla F_{k}\left(w^{\left(t,\tau \right)}\right){\left|\right|}^{2}\\ \; & =\frac{1}{\gamma }\sum_{\tau =0}^{\gamma }||\nabla F_{k}\left(w^{t}\right)-\nabla F_{k}\left(w^{\left(t,\tau \right)}\right){\left|\right|}^{2} \end{array}$$

According to proposition (A1), $C_{4}$ is further expressed as:

(A9) $$\begin{array}{cc} C_{4} & =\frac{L^{2}}{\gamma }\sum_{\tau =0}^{\gamma }\left|\right|w^{t}-w^{\left(t,\tau \right)}{\left|\right|}^{2}=\frac{L^{2}}{\gamma }{\sum_{\tau =0}^{\gamma }\eta^{2}\left|\right|\sum_{\lambda =0}^{\tau }G_{k}\left(\omega_{k}^{\left(t,\lambda \right)}\right)\left|\right|}^{2}\\ \; & =\frac{L^{2}\eta^{2}}{\gamma }\sum_{\tau =0}^{\gamma }\underset{C_{5}}{\underset{︸}{\left|\right|\sum_{\lambda =0}^{\tau }\left(G_{k}\left(\omega_{k}^{\left(t,\lambda \right)}\right)-\nabla F_{k}\left(\omega^{\left(t,\lambda \right)}\right)\right)+\nabla F_{k}\left(\omega^{\left(t,\lambda \right)}\right){\left|\right|}^{2}}} \end{array}$$

From $||a+{b||}^{2}\leq {2||a||}^{2}+{2||b||}^{2}$ and proposition (A1) and (A2), $C_{5}$ can be expressed as:

(A10) $$\begin{array}{cc} C_{5} & \leq 2\sum_{\lambda =0}^{\tau }\left|\right|G_{k}\left(\omega_{k}^{\left(t,\lambda \right)}\right)-\nabla F_{k}\left(\omega^{\left(t,\lambda \right)}\right){\left|\right|}^{2}+2\sum_{\lambda =0}^{\tau }||\nabla F_{k}\left(\omega^{\left(t,\lambda \right)}\right){\left|\right|}^{2}\\ \; & \leq 2\tau \sigma^{2}+2\sum_{\lambda =0}^{\tau }\left|\right|\left(\nabla F_{k}\left(\omega^{\left(t,\lambda \right)}\right)-\nabla F_{k}\left(\omega^{t}\right)\right)+\nabla F_{k}\left(\omega^{t}\right){\left|\right|}^{2}\\ \; & \leq 2\tau \sigma^{2}+4\sum_{\lambda =0}^{\tau }||\nabla F_{k}\left(\omega^{\left(t,\lambda \right)}\right)-\nabla F_{k}\left(\omega^{t}\right){\left|\right|}^{2}+4\tau ||F_{k}\left(\omega^{t}\right){\left|\right|}^{2}\\ \; & \leq 2\tau \sigma^{2}+4\tau L^{2}\left|\right|\omega^{\left(t,\lambda \right)}-\omega^{t}\left|\right|+4\tau ||\nabla F_{k}\left(\omega^{t}\right){\left|\right|}^{2} \end{array}$$

Integrating $\left|\right|\omega^{\left(t,\lambda \right)}-\omega^{t}{\left|\right|}^{2}$ from $C_{5}$ into $C_{4}$, we can obtain:

(A11) $$\left|\right|w^{t}-w^{\left(t,\tau \right)}{\left|\right|}^{2}=2\eta^{2}\tau \sigma^{2}+4\eta^{2}\tau L^{2}\left|\right|\omega^{\left(t,\lambda \right)}-\omega^{t}{\left|\right|}^{2}+4\eta^{2}\tau ||\nabla F_{k}\left(\omega^{t}\right){\left|\right|}^{2}$$

Transform it into:

(A12) $$\left|\right|w^{t}-w^{\left(t,\tau \right)}{\left|\right|}^{2}=\frac{1}{\left(1-4\eta^{2}L^{2}\tau \right)}\left(2\eta^{2}\tau \sigma^{2}+4\eta^{2}\tau \;||\nabla F_{k}\left(\omega^{t}\right){\left|\right|}^{2}\right)$$

$||\nabla F_{k}\left(\omega^{t}\right){\left|\right|}^{2}$ can be further derived from $C_{3}$ to obtain $C_{4}$:

(A13) $$\begin{array}{cc} C_{4} & \leq \frac{L^{2}}{\gamma }\sum_{\tau =0}^{\gamma }\frac{1}{\left(1-4\eta^{2}L^{2}\tau \right)}\left(2\eta^{2}\tau \sigma^{2}+4\eta^{2}\tau ||\nabla F_{k}\left(\omega^{t}\right){\left|\right|}^{2}\right)\\ \; & \leq \frac{L^{2}}{\gamma }\sum_{\tau =0}^{\gamma }\frac{1}{\left(1-4\eta^{2}L^{2}\tau \right)}(2\eta^{2}\tau \sigma^{2}+8\eta^{2}\tau (||\nabla F\left(w^{t}\right){\left|\right|}^{2}+A\sum_{k=1}^{N}d_{k}+B)) \end{array}$$

Let $H=N\sum_{k=1}^{N}\left|\right|n_{k}-p_{k}{\left|\right|}^{2}$, $K=\frac{L^{2}}{\gamma }\sum_{\tau =0}^{\gamma }\frac{2\eta^{2}\tau }{\left(1-4\eta^{2}L^{2}\tau \right)}$; then, $C_{2}$ can be expressed as:

(A14) $$\begin{array}{cc} C_{2} & \leq 4H(||\nabla F\left(w^{t}\right){\left|\right|}^{2}+B)+\frac{4AH}{N}\sum_{k=1}^{N}d_{k}\\ \; & +2\sum_{k=1}^{N}p_{k}K(\sigma^{2}+4(||\nabla F\left(w^{t}\right){\left|\right|}^{2}+A\sum_{k=1}^{N}d_{k}+B))\\ \; & \leq 4H(||\nabla F\left(w^{t}\right){\left|\right|}^{2}+B)+\frac{4AH}{N}\sum_{k=1}^{N}d_{k}+\\ \; & +2K(\sigma^{2}+4(||\nabla F\left(w^{t}\right){\left|\right|}^{2}+A\sum_{k=1}^{N}p_{k}d_{k}+B)) \end{array}$$

Substituting $C_{2}$ into $C_{1}$ gives:

(A15) $$\begin{array}{cc} C_{1}= & \frac{1-4(H+2K)}{2}||\nabla F\left(w^{t}\right){\left|\right|}^{2}+\frac{1}{2}\left|\right|\sum_{k=1}^{N}p_{k}G_{k}{\left|\right|}^{2}\\ \; & -2HB-\frac{2AH}{N}\sum_{k=1}^{N}d_{k}-K\left(\sigma^{2}+4\left(A\sum_{k=1}^{N}p_{k}d_{k}+B\right)\right) \end{array}$$

Integrate into:

(A16) $$\begin{array}{cc} \; & F\left(w^{t+1}\right)-F\left(w^{t}\right)\leq -\eta [\frac{1-4(H+2K)}{2}||\nabla F\left(w^{t}\right){\left|\right|}^{2}\\ \; & -2HB-\frac{2AH}{N}\sum_{k=1}^{N}d_{k}-K\left(\sigma^{2}+4\left(A\sum_{k=1}^{N}p_{k}d_{k}+B\right)\right)]\\ \; & +\frac{\eta (L\eta -1)}{2}\left|\right|\sum_{k=1}^{N}p_{k}G_{k}{\left|\right|}^{2} \end{array}$$

when Lη−1<0, i.e., η<1/L, we can obtain:

(A17) $$\begin{array}{cc} F\left(w^{t+1}\right)-F\left(w^{t}\right) & \leq -\eta [\frac{1-4(H+2K)}{2}{\left||\nabla F\left(w^{t}\right)|\right|}^{2}-2HB\\ \; & -\frac{2AH}{N}\sum_{k=1}^{N}d_{k}-K\left(\sigma^{2}+4\left(A\sum_{k=1}^{N}p_{k}d_{k}+B\right)\right)] \end{array}$$

Translocation and according to proposition (A2) and (A3):

(A18) $$\begin{array}{cc} \min_{t}\;E||\nabla F\left(w^{t}\right){\left|\right|}^{2}= & \frac{1}{T}\sum_{t=0}^{T}E||\nabla F\left(w^{t}\right){\left|\right|}^{2}\\ \; & \leq \frac{2}{1-4(H+2K)}[\frac{1}{\eta T}\left(F\left(w^{0}\right)-F_{\inf}\right)+2HB\\ \; & +\frac{2AH}{N}\sum_{k=1}^{N}d_{k}+K\left(\sigma^{2}+4\left(A\sum_{k=1}^{N}p_{k}d_{k}+B\right)\right)] \end{array}$$

which completes the proof. □

## Appendix B

**Proof of Lemma A1.** To simplify the analysis, the convergent bounds in Theorem 1 are represented as five terms, i.e, $N_{1}=\frac{2}{1-4(H+2K)}$, $N_{2}=\frac{1}{\eta T}\left(F\left(w^{0}\right)-F_{\inf}\right)$, $N_{3}=2HB$, $N_{4}=\frac{2AH}{N}\sum_{k=1}^{N}d_{k}$, $N_{5}=K\left(\sigma^{2}+4\left(A\sum_{k=1}^{N}p_{k}d_{k}+B\right)\right)$, where $H=N\sum_{k=1}^{N}\left|\right|n_{k}-p_{k}{\left|\right|}^{2}$, and it is evident that it consists of four parts related to the aggregation weights $p_{k}$. When there is a greater disparity between the dataset sizes $n_{k}$ and the aggregation weights $p_{k}$, the value of *H* increases, leading to larger values for $N_{3}$ and $N_{4},$ which can often amplify the bounds. From $N_{4}$ and $N_{5}$, we can observe that having different $d_{k}$ values might help in reducing the bounds. To minimize the bounds, we consider transforming the convergence bound expression, $N_{2}+N_{3}+N_{4}+N_{5}/N_{1},$ into $N_{2}+N_{3}+N_{4}+N_{5}-\varepsilon N_{1}$, and then we tackle the following optimization problem to achieve minimization:

(A19) $$\begin{array}{cc} Y(p_{k})= & \frac{1}{\eta T}\left(F\left(w^{0}\right)-F_{\inf}\right)+2HB+\frac{2AH}{N}\sum_{k=1}^{N}d_{k}+\\ \; & K\left(\sigma^{2}+4\left(A\sum_{k=1}^{N}p_{k}d_{k}+B\right)\right)-\frac{1-4(H+2K)}{2}\varepsilon \end{array}$$

where $\sum_{k=1}^{N}p_{k}=1$ and $p_{k}>0$. For further optimization, we can set the derivative of $Y(p_{k})$ to zero to achieve the minimization, as follows:

(A20) $$\begin{array}{c} Y^{\prime }\left(p_{k}\right)=4BN\left(n_{k}-p_{k}\right)+4A\left(n_{k}-p_{k}\right)\sum_{k=1}^{N}d_{k}+4Ad_{k}+4\varepsilon N\left(n_{k}-p_{k}\right)=0 \end{array}$$

Consolidating, we obtain:

(A21) $$p_{k}=n_{k}+\frac{Ad_{k}}{BN+A\sum_{k=1}^{N}d_{k}+\varepsilon N}$$

This leads to:

(A22) $$p_{k}\propto n_{k}+Jd_{k}$$

*J* is a constant greater than zero. which completes the proof. □
